# Connectivity and circuitry in a dish versus in a brain

**DOI:** 10.1186/s13195-015-0129-y

**Published:** 2015-06-04

**Authors:** Vorapin Chinchalongporn, Peter Koppensteiner, Deborah Prè, Wipawan Thangnipon, Leonilda Bilo, Ottavio Arancio

**Affiliations:** Department of Pathology & Cell Biology, Columbia University, New York, NY 10032 USA; Taub Institute for Research on Alzheimer’s Disease and the Aging Brain P&S Bldg, Room 12-420D, Columbia University, New York, NY 10032 USA; Columbia Stem Cell Initiative, CUMC, New York, NY 10032 USA; Research Center for Neuroscience, Institute of Molecular Biosciences, Mahidol University, Salaya, Nakhonpathom 73170 Thailand; Department of Neurophysiology and Neuropharmacology, Center for Physiology and Pharmacology, Medical University of Vienna, 1090 Vienna, Austria; Department of Neurosciences, Reproductive and Odontostomatological Sciences, Federico II University of Naples, 80131 Naples, Italy

## Abstract

In order to understand and find therapeutic strategies for neurological disorders, disease models that recapitulate the connectivity and circuitry of patients’ brain are needed. Owing to many limitations of animal disease models, *in vitro* neuronal models using patient-derived stem cells are currently being developed. However, prior to employing neurons as a model in a dish, they need to be evaluated for their electrophysiological properties, including both passive and active membrane properties, dynamics of neurotransmitter release, and capacity to undergo synaptic plasticity. In this review, we survey recent attempts to study these issues in human induced pluripotent stem cell-derived neurons. Although progress has been made, there are still many hurdles to overcome before human induced pluripotent stem cell-derived neurons can fully recapitulate all of the above physiological properties of adult mature neurons. Moreover, proper integration of neurons into pre-existing circuitry still needs to be achieved. Nevertheless, *in vitro* neuronal stem cell-derived models hold great promise for clinical application in neurological diseases in the future.

## Introduction

The complexity of the human central nervous system and its inaccessibility to direct studies make its modeling necessary in order to investigate physiological and pathological processes occurring in it. Animal disease models have been introduced to study pathophysiological processes and eventually develop new treatments. However, the use of animal models has drawbacks, including high costs of maintenance and difficulties to fully mimic the characteristics of a human neurological disease. *In vitro* models using patient-derived cells are currently emerging to study neuropathologies and test possible treatments, as the *in vitro* system is more scalable, controllable and cheaper. In particular, recently developed techniques to generate human induced pluripotent stem cells (iPSCs) [1] allow the investigation of cells derived from patients. This technological development has led to the need to confirm functionality of the newly generated neurons with respect to electrophysiological properties of individual neurons, their ability to express pathophysiologically relevant phenotypes, and their capability to functionally integrate into the brain’s circuitry. Despite the attractive possibility of studying newly generated human neurons, previous studies have revealed problems regarding the maturation of the stem cell-derived neurons, as well as the survival of implanted iPSC-derived cells, the directed differentiation into certain cell types [2] and the tumorigenic potential of incompletely differentiated iPSCs [2, 3]. Such limitations will have to be overcome before newly generated human neurons become clinically useful.

In this review, we will first discuss the characteristics of the development of both basic electrophysiological properties in maturing neurons and their synaptic activity, as well as integration of individual neurons into synaptic circuitry. The passive and active membrane properties and the presence of spontaneous postsynaptic currents are strong indicators of neuronal maturation and can be used to evaluate the potential therapeutic viability of the different protocols. Next, we will evaluate the derangement of synaptic properties underlying disease processes. Finally, we will discuss recent studies on stem cell-derived human neurons and how they recapitulate physiopathological features of brain neurons.

## The physiological role of synaptic neurotransmission

### Electrophysiological markers of neuronal development and stem cell conversion

A central characteristic of neurons is their ability to send and receive signals by means of action potential (AP) formation and propagation with subsequent synaptic neurotransmission. The underlying neuronal properties permitting intercellular signaling are progressively changing during early network formation as well as during differentiation of stem cells into neurons. Indeed, the developmental stage of neurons can be assessed electrophysiologically by measuring their passive and active membrane properties as well as synaptic currents. Passive membrane properties commonly investigated in studies monitoring neuronal development include input resistance (R_in_), membrane capacitance (C_m_), and the membrane time constant (τ) as well as the resting membrane potential (RMP). With progressive neuronal development, R_in_ and τ values have been found to decrease whereas C_m_ values increase and the RMP shows a negative shift [4, 5]. These passive membrane properties render immature neurons highly excitable, as high R_in_ and τ values together with depolarized RMPs enable AP generation in response to weak membrane currents. Thereby, the electrophysiological profile of immature neurons might function to compensate for the rather low frequencies of synaptic neurotransmission in early developing networks by increasing the chance of AP generation upon presynaptic transmitter release. Similar to passive membrane properties, measurement of active membrane properties underlying AP formation and propagation allows for analysis of the electrophysiological profile of developing neurons and is particularly helpful in distinguishing pyramidal glutamatergic from inhibitory interneurons via their distinct AP shapes and firing patterns [6].

Synaptic activity is another fundamental feature that characterizes neurons. The activity in early developing networks differs from that of mature networks by a number of factors, including the excitatory–inhibitory shift of γ-aminobutyric acid (GABA), the occurrence of giant depolarizing potentials (GDPs) and progressively increasing frequencies of both GABAergic and glutamatergic spontaneous neurotransmission, indicative of developmental synaptogenesis [7]. Importantly, spontaneous synaptic activity after birth serves as a guidance signal for synaptogenesis in immature neurons (reviewed in [8]). Although the progression of synaptic neurotransmission over the course of iPSC-derived neuron differentiation has been described recently [9, 10], the excitatory–inhibitory shift of GABA and the importance of GDPs have yet to be investigated in detail. Thus, it is currently unknown whether neuronal differentiation from somatic cells shares the electrophysiological characteristics of natural neuronal development.

Both in the developing and the adult central nervous system, glial cells facilitate differentiation and maturation of cells into neurons [11] and play fundamental roles in synaptic development, homeostasis and activity [12]. Astrocytes promote developmental synaptogenesis via the release of thrombospondins, hevin and secreted protein acidic and rich in cysteine [13, 14]. The expression of thrombospondins coincides with the early postnatal period of synaptogenesis while hevin and secreted protein acidic and rich in cysteine are also expressed in astrocytes in the adult central nervous system [15, 16]. In their role of regulating the synaptic development and maintenance, the astrocytes act by releasing glutamate or ATP [17, 18] and cell contact molecules such as ephrins [19,20]. Consistently, co-cultures of neural progenitor cells (NPCs) derived from human iPSCs together with astrocytes show significantly faster rates of neuronal maturation [10]. Additionally, microglial cells, in coordination with the complement system, are involved in synapse degradation, termed synaptic pruning [21–23]. Although several studies utilizing iPSC-derived neurons used glial-conditioned medium obtained from astrocyte-rich cultures, the lack of microglial cells in iPSC-derived neuronal cultures might negatively impact synaptic development in such cells. Additionally, since microglia cells require complement system activation to exert their function on synaptic pruning, the addition of complement system proteins might be required.

### Synaptic plasticity and its molecular machinery

Strong, repetitive synaptic activity leads to synaptic plasticity. Long-term potentiation (LTP) and long-term depression (LTD) are two forms of synaptic plasticity that have been widely investigated and are linked to learning and memory (reviewed in [24]). During induction of LTP at the CA3–CA1 hippocampal synapse, large amounts of glutamate are released from the presynaptic terminal and bind to the *N*-methyl-d-aspartate receptors (NMDARs) and the α-amino-3-hydroxy-5-methyl-4-isoxazolepropionic acid receptors located at the postsynaptic membrane. While during the resting state NMDARs are blocked by Mg^2+^ ions, when a strong depolarization is induced by glutamate binding to α-amino-3-hydroxy-5-methyl-4-isoxazolepropionic acid receptors the Mg^2+^ block is removed and Ca^2+^ ions enter the cell via NMDARs. The calcium influx activates a cascade of second messenger events that trigger nuclear transcription factors followed by gene transcription, protein synthesis underlying synaptic strengthening. NMDAR-dependent LTP thus requires presynaptic glutamate release and postsynaptic depolarization promoting structural presynaptic and postsynaptic changes [25]. Conversely, LTD can be induced by low-frequency stimulation of presynaptic terminals, minimizing postsynaptic calcium influx through NMDARs and the preceding depolarization. Low levels of NMDAR activation promote long-lasting decreases in synaptic efficacy. Importantly, LTD might involve endocytosis of synaptic α-amino-3-hydroxy-5-methyl-4-isoxazolepropionic acid receptors and reduction of dendritic spine surfaces [26].

In summary, important factors that control synaptic potentiation or depression are the intensity of presynaptic activation, postsynaptic depolarization and the amount of calcium flowing through the NMDARs [27]. Because of the relevance of LTP and LTD in memory formation, complete neuronal maturation includes the formation of synapses capable of undergoing synaptic plasticity. Currently, there have been no studies reporting functional synaptic plasticity in iPSC-derived or direct conversion-derived neurons. Obtaining neurons capable of undergoing synaptic plasticity will probably constitute one of the most challenging tasks in the field of stem cell research.

### Methods to analyze neuronal properties *in vitro* and *in vivo*

In order to investigate the functionality of the neurons, different methodologies have been developed over the years. The method of choice depends on many factors: the level of resolution needed, the possibility to study neuronal circuitry or intact systems, the necessity to manipulate the experimental conditions, the interest in studying synaptic events or sensory responses and, no less important, the cost, time and difficulty of using the technique. Most of the time, complete understanding of a phenomenon requires the use of more than one methodology. In this chapter, we present the methods most frequently used by neuroscientists to analyze neuronal properties. A summary of the characteristics of the methods described in this section is presented in Table 1.

**Table 1:** Summary of methods to analyze neuronal properties

**Method of study**	**Single cell resolution**	**Allowing the recording of multiple single cell signals**	**Possibility to study intact systems**	**Analysis of brain circuitry**	**Possibility to manipulate the experimental conditions (that is, drug concentration)**	**Difficulty in performing the experiment/time cost**	**Possibility to study synaptic events**	**Possibility to analyze sensory responses**	**Invasivity**	**Cost**
*In vitro* intracellular recording	+	–	–	–	+	–	+	–	+	–
Intracellular recording on acute brain slices	+	–	–	–	+	+	+	–	+	–
Extracellular recording of local field potential	–	–	–	+	–	–	–	–	–	–
Multi-electrode array recording	+	+	–	–	–	–	+	–	–	+
*In vivo* recording	–	–	+	–	–	+	–	+	+	–
Calcium imaging	–	+	–	–	+	–	+	–	–	+
Optogenetic	+	+	+	+	+	+	+	+	+	+
Vesicle cycling	–	–	–	–	+	+	+	–	+	–

Different aspects are taken into consideration for the methods described (marked + or – depending on the existence of the characteristic described)

#### Electrophysiology

Electrophysiological properties of neurons *in vitro* and *in vivo* can be studied by a series of techniques including both intracellular and extracellular recordings, as well as imaging techniques allowing investigation of neurotransmitter release. The method of choice depends on the required level of detail and the specific scientific question to be answered. At a cellular level, intracellular recordings are used to measure voltages or currents across the cell membrane [28]. The patch clamp technique [29] allows the study of single or multiple ion channels (excised patch technique), or of the electrical properties of an entire cell (whole cell patch and perforated patch). The use of patch clamp in cell cultures enables the characterization of basic electrophysiological properties of cells under both physiological and pathological conditions and the investigation of synaptic neurotransmission. In contrast, patch clamp in brain slices enables the investigation of semi-conserved neuronal circuits, and the measurement of neurotransmission in specific areas of the brain.

At a multicellular level, extracellular recordings of local field potentials are used to study the collective activity of many cells by monitoring the signals in the extracellular space of the brain, in order to investigate the synaptic connectivity of neuronal circuits in specific areas. More recently, *in vitro* multi-electrode array methods have been developed [30] and are currently used in cell cultures or acute brain slices, with the advantage of having a high spatial and temporal resolution of signals across a neuronal network. In addition, the multi-electrode array technique can be combined with intracellular recordings [31].

Another important category of electrophysiological analysis is represented by *in vivo* recordings, where the same techniques explained above are used in live animals. With this method, one can study brain regions or neurons in their intact state with their normal complement of inputs and targets. The cells being studied usually have not been severed or damaged, in contrast to studies in brain slices, and have developed normally in the intact organism, in contrast to the culture preparation. Importantly, this technique allows for the investigation of neuronal responses to sensory stimuli.

#### Calcium imaging

Calcium is an essential intracellular messenger in mammalian neurons. At rest, most neurons have an intracellular calcium concentration of about 50 to 100 nM that can rise transiently during electrical activity to levels that are 10 to 100 times higher [32]. Use of the calcium imaging technique to investigate calcium variations in living cells and animals is thus fundamental. In presynaptic terminals, Ca^2+^ influx triggers exocytosis of synaptic vesicles containing neurotransmitters [33]. Postsynaptically, a transient rise of the Ca^2+^ level in dendritic spines is essential for the induction of activity-dependent synaptic plasticity [34].

Briefly, the calcium imaging technique consists of two steps: loading the cells with fluorescent molecules that can respond to the binding of Ca^2+^ ions by changing their fluorescence properties, and detecting Ca^2+^ transients using a fluorescence microscope and a charge-coupled device camera. Images are then analyzed by measuring fluorescence intensity changes, and the derived fluorescence intensities are plotted against calibrated values for known calcium levels to learn the Ca^2+^ concentration.

There are two main classes of calcium indicators: chemical indicators and protein-based genetically encoded calcium indicators. Among the chemical indicators, the most recently developed Oregon Green BAPTA and fluo-4 dye families [35] are widely used in neuroscience because they are relatively easy to implement and provide large signal-to-noise ratios. Genetically encoded calcium indicators do not need to be loaded onto cells, but instead the genes encoding for these proteins can be transfected into cell lines [36]. To detect the fluorescence changes with high spatial and temporal resolution, the calcium imaging technique takes advantage of the development of new imaging instrumentation. A major advance in the early 1990s was the introduction of two-photon microscopy by Denk and colleagues [37] and its use for calcium imaging in the nervous system [38]. In a recent study, the group of Deisseroth developed a technique, termed fiber photometry, to measure Ca^2+^ signals in neuronal processes *in vivo* using the genetically encoded calcium indicator GCaMP [39]. This method might also be useful to monitor neuronal activity of *in vitro* differentiated neurons following transplantation in future studies.

#### Optogenetics

Recently, a new technology has been developed to control, either by exciting or inhibiting, the activity of targeted individual neurons: optogenetics. An August 2005 report described how neurons became precisely responsive to light upon introduction of a microbial opsin gene [40]. In the following years, several additional reports demonstrated that some microorganisms known for being able to produce visible light-gated proteins that directly regulate the flow of ions across the plasma membrane (channelrhodopsin, bacteriorhodopsin and halorhodopsin) are capable of turning neurons on or off, rapidly and safely, in response to diverse colors of light [41–43]. This innovative approach allows neuroscientists to control defined events in defined neuronal types and projections at defined times in intact systems, while maintaining high temporal (AP scale) precision. An important application of optogenetics is represented by its use for studying well-defined biochemical events within behaving mammals [44–46]. The potential of optogenetics selectively activating specific neuronal subtypes has also been applied to study pathological processes. Gradinaru and colleagues used optogenetics to systematically drive or inhibit an array of distinct circuit elements in freely moving parkinsonian rodents and found that therapeutic effects within the subthalamic nucleus can be accounted for by direct selective stimulation of afferent axons projecting to this region [47].

Importantly, optogenetic approaches have been used to confirm functional integration into pre-existing networks of neurons and NPCs differentiated from iPSCs and embryonic stem cells (ESCs) [48–52]. By selectively expressing channel rhodopsin in iPSC-derived or ESC-derived neurons, functional synaptogenesis can be confirmed by measuring postsynaptic responses triggered by light-stimulation in co-cultured mouse neurons as well as in mouse brain slices following stereotaxic injection of *in vitro* differentiated cells. Furthermore, a recent study transplanted ESC-derived motor neurons that formed functional neuromuscular connections allowing for optogenetic control of muscle contraction *in vivo* [51]. The use of optogenetic technologies in combination with *in vitro* differentiation methods thus provides an attractive prospect for future patient-specific treatments.

#### Vesicle cycling

Neurotransmitter release is often investigated though FM dye visualization during vesicle cycling [16]. These dyes are fluorescent molecules used to monitor the movement of synaptic vesicles. Their use is based on their amphipathic properties. Dyes can be reversibly endocytosed and exocytosed via lipid membrane budding and fusion, but dyes themselves cannot permeate the membrane. To image presynaptic function, FM dyes are taken up into synaptic vesicles in an activity-dependent manner, termed loading. After washout and subsequent chemical/electrical stimulation, dyes are released from presynaptic boutons, termed unloading. The loss in fluorescence intensity during loading and unloading steps thus provides information about phenotypes and rates of presynaptic neurotransmission [53].

### Clinical investigation of neuronal function

The study of neuronal electrophysiological properties in a clinical setting has been mostly permitted by electroencephalography (EEG) and evoked potentials. Nowadays, the combination of scalp EEG recording with functional magnetic resonance imaging (fMRI) allows an in-depth understanding of phenomena underlying EEG activity. By combining the two techniques, one can determine which brain areas change their neuronal activity during an epileptic discharge, and consequently understand the anatomical location and physiological activation of neuronal networks generating normal and abnormal EEG signals. For instance, in temporal lobe epilepsy, temporal localized spiking on EEG has been found to be accompanied by a widespread activation in distant regions on fMRI, especially in the contralateral homologous regions, suggesting that the neuronal network involved in temporal spiking is much wider than what appears on scalp EEG [54]. Furthermore, in idiopathic generalized epilepsies, the bilateral and synchronous spike and wave pattern on EEG is accompanied on fMRI by activation in the thalamus and by widespread, bilateral and symmetrical deactivation in the cortex.

Nowadays, clinical investigation also uses techniques that have been developed especially for animal studies. For instance, at the level of single neurons, epileptic activity is investigated on brain specimens resected during epilepsy surgery using the patch clamp technique. These studies have shown a major role for ion channels in the generation of excessive and hypersynchronous neuronal discharges (the so-called paroxysmal depolarizing shifts), a hallmark of the epileptic activity. In fact, the abovementioned discharges originate from alterations of neuronal excitability or synaptic transmission induced by spontaneous or experimental modifications of the properties of voltage-dependent or receptor-activated ion channels [55].

Integration of stem cell-derived neurons is not yet used in clinical practice. However, implants of iPSC-derived neuronal precursor cells in embryonic or adult mouse brains are currently explored. Thus, one can envision a time at which EEG, fMRI and evoked potentials will be utilized to assess functionality of stem cell-derived neuronal grafts in humans.

## Synaptic dysfunction in neurological diseases

### On the abnormality of neuronal circuits in neurological diseases

Dysfunctional synaptic circuits have been found in neurodevelopmental disorders such as autism spectrum disorders, psychiatric disorders such as schizophrenia, and neurodegenerative disorders including Alzheimer’s disease (AD), Parkinson’s disease (PD) and spinal muscular atrophy (SMA) [56–66]. Both hyperconnectivity within local and short-distance frontal cortical connectivity, and hypoconnectivity between frontal lobe and temporal lobe multimodal association cortices have been described in autism spectrum disorder human brain [56]. In schizophrenia patients, massive gray matter loss results in the reduction of spine density in the superior temporal gyrus associated with auditory hallucinations [57]. In addition, a mouse model of NMDAR hypofunction can reproduce the hippocampal deficits and cognitive abnormalities that have been observed in schizophrenia patients [58].

In AD amyloid precursor protein transgenic mice there is a reduction of sodium current density, which results in aberrant EEG activity and impaired performance with the Morris water maze memory test [59]. Electroencephalographic recordings in human amyloid precursor protein transgenic mice revealed a network hypersynchrony caused by decreased levels of the interneuron-specific and parvalbumin cell-predominant voltage-gated sodium channel subunit Nav1.1 [60]. Aberrant increases in network excitability and compensatory inhibitory mechanisms may contribute to amyloid beta-induced neurological deficits in human amyloid precursor protein mice and, possibly, also in humans with AD [61, 62]. Massive destruction of the dopaminergic nigrostriatal circuit entails motor and cognitive complications in PD patients [63]. Mutant huntingtin expression in Huntington’s mouse model changes striatal excitatory synaptic activity by decreasing glutamate uptake and increasing signaling at NMDAR [64]. In Sod1 transgenic mice, an amyotrophic lateral sclerosis model, motor neurons become hyperexcitable. Diminished control of motor neuron firing has been explained by the loss of presynaptic motor axon input on Renshaw cells occurring in the early stages of amytrophic lateral sclerosis and the disconnection of the recurrent inhibitory circuit [65]. In a Drosophila model of SMA, chronic dysfunction of the sensory-motor circuit causes functional impairments such as muscle weakness and progressive motor neurodegeneration [66]. Taken together, observations from these models and patients might inspire studies using human iPSC-derived models.

### Role of proteins related to neurodegenerative diseases in alterations of synaptic properties and learning and memory

One of the most common features of neurodegenerative diseases is the progressive and region-specific accumulation of proteins. These aggregated proteins induce neurons in the affected regions to adapt to their immediate environment and affect synaptic plasticity [67]. For instance, two proteins are involved in AD: amyloid beta, which is deposited in extracellular amyloid plaques; and tau, forming intracellular neurofibrillary tangles. Amyloid beta inhibits NMDAR-dependent LTP and promotes LTD in the hippocampus. Additionally, dysregulation of LTD is associated with GSK-3β, an enzyme responsible for hyperphosphorylation of tau [68].

With respect to PD, cortical Lewy bodies are found in the soma of nigral dopamine neurons. These Lewy bodies are composed of protofibrils of aggregated α-synuclein [69]. In animal models overexpressing mutated forms of α-synuclein, dopaminergic neurotransmission is impaired, leading to loss of dopaminergic neurons and the impairment of LTD in medium spiny striatal neurons and ultimately to cognitive and motor deficits.

With regard to SMA, a deficiency of survival motor neuron protein is associated with sensory-motor network dysfunction and muscle deterioration. Importantly, in mice lacking survival motor neuron protein a reduction of proprioceptive synaptic inputs onto motor neurons and spinal reflex deficits have been observed [70].

Nevertheless, animal models cannot fully recapitulate the key aspects of human models [71]. Regarding tauopathy, the human adult central nervous system can express six different tau isoforms whereas only tau-4R is expressed in mice adult neurons. Additionally, an important concern is the failure of patient-specific donor cells to maintain age-associated markers in late-onset human iPSC-based disease models. Expression of progerin, a truncated form of lamin A involved in Hutchinson–Gilford progeria syndrome, has been studied as a strategy for reintroducing age-like features such as age-related markers in iPSC fibroblasts and robust degenerative phenotypes [72, 73]. Thus, it would be interesting to test whether low levels of progerin could facilitate iPSC maturation [74].

## Functional neuronal circuits in a dish: what we have found from stem cell research

### Physiological neuronal development: analysis of neural markers

Neuronal differentiation of stem cells is commonly monitored through immunocytochemical methods confirming the expression of specific neuronal markers [1]. For instance, human iPSCs commonly express pluripotent markers including human embryonic stem cell surface antigens SSEA3 and SSEA4, tumor-related antigens TRA-1-60, TRA-1-81 and TRA-2-49/6E, and NANOG protein. Undifferentiated pluripotent stem cell marker genes OCT3/4 and SOX2 are also detected by RT-PCR. The conversion of reprogrammed iPSCs into neuronal cells can be confirmed using the neuroectodermal marker nestin, the neuronal lineage marker Pax6, the neuronal differentiation marker T-box brain 1, and layer-specific markers for developing cortical cells including upper layer (cut-like homeobox 1) and deeper layer projection neuron markers (COUP-TF-interacting protein 2) [75]. Similar neuronal markers can also be observed in pyramidal neurons derived from ESCs [76]. Regional identity is, in turn, specified through Forkhead box G1 and Orthodenticle homeobox 2 for the dorsal forebrain region, whereas distal-less homeobox 2 is a marker for developing ventral forebrain identity in reprogrammed and embryonic-derived neurons [77, 78]. Mature reprogrammed neurons can be identified through microtubule-associated protein MAP2 expression [79]. In addition, neuorotransmitter-related markers are used to indicate fully developed reprogrammed forebrain neurons. Markers for glutamatergic neurons include the vesicular glutamate transporter VGLUT1 and phosphate-activated glutaminase. Glutamate decarboxylase is a commonly used marker to identify GABAergic neurons differentiated from human iPSCs [80]. Immunocytochemical markers have also been used to investigate synapse formation in iPSC-derived neurons. Among these markers are synaptophysin and synapsin I on the presynaptic side and postsynaptic density protein PSD95 as well as Homer1 at the postsynaptic level [75].

Relying on immunocytochemical methods alone to identify synaptic connections might have significant pitfalls. Postsynaptic receptors have been shown without corresponding markers for the presynaptic active zone [81]. Similarly, presynaptic clusters without corresponding postsynaptic proteins have been identified [82]. In other studies, areas with both presynaptic and postsynaptic proteins have revealed no functional synapses [83]. On the other hand, it has been demonstrated that synapsin I might not always be present in functional synapses [84]. Furthermore, nonspecific antibody staining might yield spurious results [85]. Thus, it has been suggested that industrial antibody producers should be motivated to test their products more rigorously before making them commercially available to the scientific community [86].

Various studies characterized the presynaptic expression of neurotransmitters as well as the postsynaptic expression of the respective neurotransmitter receptors, such as glutamate receptors and GABA receptors [78, 87–90]. However, prominent expression of such markers was not always reflected in equally prominent and mature synaptic neurotransmission. In the study by Vazin and colleagues, iPSC-derived cortical neurons readily expressed high levels of GABA and glutamate, but electrophysiological investigation showed highly immature synaptic currents at low frequency and devoid of typical neurotransmission-derived current kinetics [78]. Correspondingly, the study by Marchetto and colleagues reported altered spontaneous neurotransmission in iPSC-derived neurons of Rett syndrome patients compared with wild type but did not find alterations in the immunohistochemical analysis of synaptic markers [87]. In contrast, a study on iPSC-derived neurons of trisomy 21 patients found a reduction in spontaneous neurotransmission frequency and reduced synapsin I staining [90]. In iPSC-derived neurons of schizophrenia patients, various presynaptic and postsynaptic markers appeared to be aberrantly expressed, but measured neuronal connectivity remained unaffected [88, 91]. Similarly, in a study of spontaneous movement disorder patient-derived neurons, reductions in synaptic neurotransmission frequency and amplitude correlated with reduced glutamate receptor and synapsin I expression. The localization of synaptic proteins alone is thus insufficient and the use of electrophysiological methods is necessary to confirm the presence of functional synapses or pathophysiologically related alterations.

Recent iPSC studies are using additional approaches, such as microarray analysis, deep RNA sequencing, and quantitative PCR arrays, to determine the status of neuronal differentiation [92]. Microarray analysis offers a customized array containing genes specific to particular cell populations or signaling pathways. This method can assess characteristic features of the pluripotent state and stem cell differentiation. Deep RNA sequencing provides high sensitivity to detect low-copy transcripts, long noncoding RNAs, novel transcripts, and splice isoforms which are seen during the transition from pluripotent stem cells to early differentiated neurons [93]. Using a quantitative PCR array, the expression of key pluripotency-associated genes in the iPSC differentiation can be quantified and the dynamic of interested gene expression can be tracked [94]. Nevertheless, it should be pointed out here that the presence of neural markers alone is insufficient to confirm full neuronal functionality [9]. Thus, analysis of electrophysiological parameters is to be performed when studying *in vitro* differentiated neurons.

### A comparison of electrophysiological and genetic profiles of induced pluripotent stem cell-derived and directly converted neurons

Terminally differentiated cells can be converted into neurons either by first generating iPSCs [1] and subsequent neuronal differentiation [9, 87, 95, 96] or by direct neuronal conversion using viral transduction of specific factors to produce induced neuronal (iN) cells [97–101]. These methods allow for the generation of a range of neuronal cell types, including dopaminergic neurons [100], motor neurons [96], cortical neurons [102] and sensory neurons [103]. Both methods of neuronal differentiation produce glutamatergic pyramidal neurons [9, 99, 101, 104] as well as GABAergic interneurons [105, 106]. In many of these studies, electrophysiological methods were used to investigate the functional quality of generated neuronal cells.

#### Electrophysiological development *in vivo*

In the past three decades, the development of electrophysiological properties *in vivo* has been well characterized in rodent neurons of various brain structures [5, 107–110]. It was found that neurons in early neonatal development are characterized by R_in_ values higher than 1 GΩ, with a rapid decline over the course of the first postnatal month to around 100 MΩ. Similarly, τ values are usually high (>30 milliseconds) around the perinatal period, indicating slow adaptation of the membrane potential to membrane currents. τ values also decrease rapidly in the first postnatal month to around 10 to 20 milliseconds. C_m_ values have been shown to be below 50 pF at the time of birth and progressively increase over the first 2 weeks after birth. Consistently, APs of perinatal rodent neurons are characterized by small amplitudes of 30 to 40 mV, broad full widths at half-maximum (FWHMs) of more than 3 milliseconds and depolarized threshold potentials of around −30 mV. Similar to passive membrane properties, AP properties mature equally rapid over the first postnatal month, with AP amplitudes increasing to >90 mV, FWHMs becoming faster than 1.5 milliseconds and AP thresholds reaching more hyperpolarized values at around −40 mV, depending on the specific brain area and cell type. In addition to intrinsic electrophysiological properties, the development of synaptic neurotransmission has been equally well described in various studies using developing rodent neurons. It was shown that neurotransmission events occur already in the immediate neonatal period at low frequency (<0.5 Hz) and that the frequency of spontaneous neurotransmission increases progressively with development [111, 112]. An indication for physiological network formation is the occurrence of GDPs, which are network-wide bursts of synaptic activity during the first postnatal week in rodents [7]. GDPs are associated with excitatory actions of GABA and thus occur in early neuronal development prior to the excitatory–inhibitory shift.

In contrast to rodents, there are only a few studies on the development of electrophysiological properties in primate neurons. The stage of rodent brain development is thought to be equivalent to the developmental stage of the human brain around the time of the third trimester of pregnancy [113]. Consistently, a study in nonhuman primates found that neurons at the time of birth present electrophysiological and synaptic properties that are almost fully matured [114]. Thus, although the level of neuronal maturity at the time of birth is different between rodents and primates, including humans, it is likely that neurophysiological properties develop in a similar manner across different species, although with different time courses.

#### Electrophysiological development of induced pluripotent stem cell-derived neurons

Neurons derived from either rodent or human iPSCs have been shown to fire a single AP in response to depolarizing current injections as early as 3 weeks after differentiation, and the number of generated APs rises with increased culture durations [9, 10, 106]. Table 2 presents an overview of recent electrophysiological characterizations of human iPSC-derived neurons. Interestingly, one study reported sodium currents in iPSC-derived neurons 1 week after differentiation, a time point at which AP firing was not yet observed [106], indicating that the presence of sodium currents alone does not implicate a cell’s ability to fire APs. In two studies on human iPSC-derived neurons, APs show amplitudes around 30 mV and FWHMs around 8 milliseconds 1 month after differentiation [9, 10]. After 2 months, AP properties matured significantly, showing AP amplitudes of 50 mV and FWHMs of 4 to 6 milliseconds. In the same studies, R_in_ values matured similarly from 1 to 3 GΩ 1 month after differentiation to 0.5 to 1 GΩ after 2 months. Other passive membrane properties followed the same path. Thus, passive membrane properties experienced similar developmental shifts as AP properties. Importantly, electrophysiological properties matured faster in human iPSC-derived neurons co-cultured with astrocytes, showing R_in_ values below 1 GΩ after 2 months [10].

**Table 2:** Summary of key studies on electrophysiological profiles of human iPSC-derived neurons

	**Pre and colleagues, 2014** [9]				**Tang and colleagues, 2013** [10]				**Zhang and colleagues, 2013** [120]
Method	Human iPSC neuronal differentiation via smad inhibition				Human iPSC-derived NPC neuronal differentiation				Human iPSC-iN cells (via Neurogenin-2 expression)
Cell line	7889O				WT126, WT33				Not stated
DIV	31 to 38	41 to 45	55	55	42	60	42	60	21
AP firing (% of cells)	20	48	60	86	NQ	NQ	NQ	NQ	>95
Single (%)	NQ	NQ	NQ	NQ	NQ	NQ	NQ	NQ	NQ
Repetitive (%)	NQ	NQ	NQ	NQ	16		73		NQ
Spontaneous AP (%)	NQ	NQ	NQ	NQ	NQ	NQ	NQ	NQ	NQ
*n*	18	27	20	15	25	NQ	22	NQ	33 to 34
R_in_ (MΩ)	2,500.0	2,200.0	1,700.0	NQ	NQ	695.0	NQ	302.0	NQ
C_m_ (pF)	NQ	NQ	NQ	NQ	NQ	27.0	NQ	119.0	NQ
τ (milliseconds)	34.0	29.0	21.0	NQ	NQ	NQ	NQ	NQ	NQ
RMP (mV)	−35.0	−38.0	−49.0	NQ	NQ	−44.0	NQ	−59.0	NQ
*n*	89	75	103	NQ	NQ	22	NQ	23	NQ
Spontaneous activity (%)	11	16	21	27	NQ	NQ	NQ	NQ	>95
Evoked potential (%)	NQ	NQ	NQ	NQ	NQ	NQ	NQ	NQ	>95
*n*	8	6	20	15	NQ	NQ	NQ	NQ	33 to 34
Co-culture	x	Mouse glial cells	x	x	Mouse astrocytes	Mouse astrocytes	Mouse glial cells		

AP, action potential; C_m_, membrane capacitance; DIV, days *in vitro*; iN, induced neuronal; iPSC, induced pluripotent stem cell; NPC, neural progenitor cell; NQ, not quantified; R_in_, input resistance; RMP, resting membrane potential; τ, membrane time constant; x, not measured, used or stated

The formation of synaptic connections is of central importance in the estimation of functional quality of *in vitro* differentiated neurons. Electrophysiologically, the measurement of spontaneous neurotransmission provides information about the network. In neurons derived from human iPSCs, spontaneous neurotransmission occurs between 3 and 6 weeks after differentiation [9, 10, 104] and requires co-culturing of iPSC-derived neurons with mouse glial cells. When cultured in glia conditioned medium, AP-independent miniature neurotransmission occurs at low frequency in 11 % of cells 1 month after differentiation of human iPSCs [9]. When co-cultured with astrocytes, AP-dependent spontaneous neurotransmission is first observed 2 weeks after differentiation followed by continuous increases in frequency and amplitude over the course of 60 days [10]. However, data on miniature neurotransmission were not described by Tang and colleagues [10]. Importantly, GDP-like neurotransmission bursts have been reported in two studies on cultured neurons derived from human iPSCs [10, 104]. Whether human iPSC-derived neurons exhibit excitatory GABAergic neurotransmission associated with the GDP phenomenon remains unclear.

There are limited electrophysiological data available on the maturation of iPSC-derived neurons *in vivo* and transplantation of human ESC-derived and iPSC-derived neurons into rodent brains has been performed in few studies [76, 115, 116]. Human ESC-derived neurons showed mature electrophysiological properties and functional integration into the developing rodent brain, as indicated by high rates of repetitive AP firing and high frequencies of synaptic neurotransmission [76, 115]. Similarly, iPSC-derived neurons were also shown to integrate into the rodent brain and form functional synapses [116]. Nevertheless, only 1 % of cells survived in the mouse brain after 7 months in the study by Nicholas and colleagues [115] and passive and active membrane properties were not characterized in detail in any of these studies. Further investigation of *in vivo* maturation of iPSC-derived neurons is thus required.

#### Electrophysiological development of directly converted neurons

The neurophysiological development of the direct conversion of mouse-derived or neonatal human-derived somatic cells appears to follow a similar path to iPSC-derived neurons, with reports of AP firing cells as early as 8 days after transduction [99, 101, 105, 117]. However, when transducing somatic cells from adult human tissue, up to 8 weeks are required to obtain neuronal cells capable of AP generation and the number of cells that fire multiple APs remains low [97, 101, 108]. Table 3 presents an overview of recent electrophysiological characterizations of human induced neurons. In the limited number of adult human iN cells capable of firing APs, the AP properties were shown to be highly variable, with broad FWHMs (>6 milliseconds), and most cells only fired single APs [108]. Importantly, it appears that human iN cells derived from adult somatic cells are not viable for long periods of time [118], making it difficult to culture human iN neuronal cells to functional maturity. Passive membrane properties of iN cells derived from adult human tissues have often only been reported superficially [97, 118]. However, a recent study reported R_in_ values of human iN cells in the GΩ range, C_m_ values around −30 pF and an RMP of −40 mV 1 month after transfection, reflecting incomplete neuronal development [108]. Importantly, studies applying direct neuronal conversion to both neonatal and adult human somatic cells demonstrated clearly that passive membrane properties of iN cells derived from adult human tissue remain highly immature at time points at which iN cells derived from neonatal human tissue have already matured significantly [97, 99, 101, 119]. Current iN methods thus appear to be rather inefficient when applied to adult human fibroblasts.

**Table 3:** Summary of key studies on electrophysiological profiles of human induced neuronal cells

	Ambasudhan and colleagues, 2011 [97]		Caiazzo and colleagues, 2011 [119]		Pang and colleagues, 2011 [99]				Pfisterer and colleagues, 2011a [100]				Pfisterer and colleagues, 2011b [100]	Son and colleagues, 2011 [149]		Yoo and colleagues, 2011 [101]	Koppensteiner and colleagues, 2014 [108]	
Method	hiN	hiN	miN	hiN	hiN	hiN	hiN	hiN	hiN	hiN	hiN	hiN	hiN	miN	hiN	hiN	hiN	hiN
Age of fibroblast donor	Neonatal	Adult	Embryonic	>40 years	Fetal	Fetal	Neonatal	11 years	Embryonic	Fetal	Fetal	Embryonic	Adult	Embryonic	Embryonic	Neonatal	30 years	Adult
Number of cell lines	2	2	1	4	3	3	3	1	1	1	1	1	3	1	1	1	1	1
Viral factors	Brn2, Myt1l, miR-124		Ascl1, Nurr1, Lmx1a		BAM + NeuroD1				BAM + Lmx1a + FoxA2		BAM		BAM	BAM + four factors	BAM + five factors	miR-9/9*, miR-124, DAM		BAM + Zic1
Conversion efficiency (%)	4 to 8	1.5 to 11	2	3 to 5	2 to 4	2 to 4	2 to 4	NQ	5	10	13	16	1 to 4	NQ	NQ	NQ	NQ	NQ
DIV	25 to 30	30	16	18	14 to 25	34 to 35	28 to 35	21	30 to 32	30 to 32	30 to 32	30 to 32	23 to 34	NQ	NQ	35 to 56	35 to 56	21 to 28
AP firing (% of cells)	81	12	NQ	NQ	21	100	79	NQ	86	83	79	91	47	90	NQ	85	60	63
Single (%)	NQ	NQ	NQ	NQ	NQ	NQ	NQ	NQ	NQ	NQ	NQ	NQ	NQ	NQ	NQ	NQ	NQ	62
Repetitive (%)	20	NQ	NQ	NQ	NQ	NQ	NQ	NQ	NQ	NQ	NQ	NQ	NQ	NQ	NQ	77	56	7
Spontaneous AP (%)	15	NQ	81	NQ	2	0	3.0	NQ	29	0	NQ	NQ	NQ	NQ	NQ	10	NQ	0
*n*	29	25	16	8	42	5	29	NQ	7	6	14	12	60	10	NQ	27	15	27
R_in_ (MΩ)	405.0	1,067.0	1,100.0	NQ	1,770.0	1,110.0	1,500.0	x	157.0	152.0	966.0	843.0	x	NQ	NQ	2,600.0	2,800.0	1,060.0
C_m_ (pF)	30.5	35.9	10.6	NQ	16.0	25.1	23.0	x	NQ	NQ	NQ	NQ	x	NQ	NQ	16.3	22.9	33.3
τ (milliseconds)	NQ	NQ	NQ	NQ	NQ	NQ	NQ	x	NQ	NQ	NQ	NQ	x	NQ	NQ	NQ	NQ	35.4
RMP (mV)	−45.0	x	−41.8	NQ	−52.1	−59.5	−41.0	x	−45.3	−41.6	−62.4	−58.6	x	−49.5	NQ	−42.0	−31.4	−42.1
*n*	21	21	16	8	52	21	29	x	7	6	14	12	x	6	NQ	27	15	27
Spontaneous activity (%)	25	43	x	x	0	19	33	x	x	x	x	x	x	x	x	71	84	0
Evoked potential (%)	x	x	x	x	0	14	0	x	x	x	x	x	x	x	x	45	47	0
*n*	8	7	x	x	20	21	6	x	x	x	x	x	x	x	x	12 to 14	10 to 12	9 to 57
Co-culture	x	x	x	x	Mouse cortical neurons	x	x	x	x	x	x	x	x	Mouse glial cells	x			

Note the more immature electrophysiological properties of neurons converted from adult somatic cells compared with those converted from fetal or neonatal tissues. AP, action potential; BAM, Brn2, Ascl1, Myt1l; DAM, NeuroD2, Ascl1, Myt1l; C_m_, membrane capacitance; DIV, days *in vitro*; five factors, Lhx3, Hb9, Isl1, Ngn2, NeuroD1; four factors, Lhx3, Hb9, Isl1, Ngn2; hiN, human induced neuronal; miN, mouse induced neuronal; NQ, not quantified; R_in_, input resistance; RMP, resting membrane potential; τ, membrane time constant; x, not measured, used or stated

Spontaneous neurotransmission has been reported for iN cells derived from direct neuronal conversion of mouse and fetal human somatic cells [99, 117]. However, directly converted iN cells require co-culturing with mature neurons in order to display spontaneous neurotransmission. Thus, it remains unclear whether iN cells alone would be capable of functional synapse formation. Although several studies report the occurrence of neurotransmission in iN cultures derived from adult human cells [97, 118, 101], example traces showing spontaneous neurotransmission might require more in-depth analysis. One of these studies recorded spontaneous neurotransmission in pure iN cultures [97] but the presented neurotransmission events appear electrophysiologically atypical, lacking fast rise and slow decay times. Furthermore, a recent study could not observe spontaneous or evoked neurotransmission in human iN cultures [108]. Consistently, GDPs have been described in mouse and fetal human fibroblast-derived iN cells [99, 117] while no GDPs have been reported for iN cells derived from adult human cells, even after transplantation into fetal mouse brains [118].

Recently, a new approach has been developed to combine direct conversion with ESC and iPSC methods, termed ESC-iN and iPSC-iN [120]. In their study, Zhang and colleagues converted NPCs derived from human iPSCs to neurons via the transduction of Neurogenin-2. These cells show very mature electrophysiological profiles, as indicated by high rates of repetitive AP firing and high frequencies of spontaneous neurotransmission, already after 3 weeks. Furthermore, the commonly observed variability in the level of neuronal differentiation of different iPSC lines could be prevented with this single-step conversion method. Although no data on passive membrane properties were presented, the displayed current and membrane potential traces appear equivalent to those of fully mature neurons. The iPSC-iN method might thus be the currently most efficient way to differentiate human iPSCs to functional neurons.

#### Gene expression analyses of human induced pluripotent stem cell-derived and induced neurons

Over the course of neuronal development, various genes are time dependently expressed and can be analyzed to determine the state of neuronal maturation. Following early neuronal lineage determination of ESCs, NPCs express nestin [121]. During embryonic brain development in humans, neuronal precursor cells migrate to their target brain regions around the eighth gestational week, where they subsequently express layer-specific markers and develop into functional neurons [122, 123]. Analysis of such region-specific and cell type-specific gene expression thus enables the identification of neuronal subtypes and their developmental stage resulting from *in vitro* differentiation. Several studies have investigated the extent of neuronal differentiation of human iPSCs using whole genome analysis [75, 115, 124]. Brennand and colleagues compared the gene expression profile of neurons differentiated from human iPSCs with that available through the Allen Brain Atlas [124]. Interestingly, they found that the gene expression profile was most similar to human neurons around the age of the first trimester of pregnancy [124]. The investigation by Mariani and colleagues came to a similar conclusion: human iPSC-derived neurons showed gene expression profiles resembling those of human neurons at gestational weeks 8 to 10 [75]. In the case of studies on directly converted neurons, no similar analyses have been performed. However, due to the general inferiority in electrophysiological profiles of human iN cells compared with iPSC-derived neurons, one would expect an equally or even more immature gene expression profile.

### Grafting neurons derived from human stem cells

Several studies have recently characterized the functional properties of the neuronal cultures derived from human stem cells. These studies have demonstrated that neurons derived from mouse and human stem cells display neuronal-like characteristics, including voltage-gated currents and the ability to fire APs. However, the maturation of human iPSC-derived neurons appears incomplete, as indicated by their immature passive and active membrane properties [9]. Importantly, co-culturing human iPSC-derived neurons with mouse glial cells improved neuronal maturation [9, 10]. In these two studies, the electrophysiological properties of the iPSC-derived neurons co-cultured with mouse glial cells were compared with those of neurons grown on laminin-coated substrates. Both groups showed an increase in the number of cells capable of firing an AP in response to a current step for neurons co-cultured with glial cells, as well as increased spontaneous synaptic activity and a more hyperpolarized resting membrane potential. While the study by Tang and colleagues focused especially on the morphology of the forming network, by showing an increased complexity of dendritic arborization in neurons co-cultured with glial cells [10], the article by Pre and colleagues analyzed especially synaptic activity, demonstrating an increase in frequency and amplitude of the spontaneous excitatory postsynaptic currents in co-cultured neurons compared with neurons plated on laminin [9]. In conclusion, while the two groups used different differentiation protocols and analyzed neurons at different time points, both studies found enhanced neuronal maturation in cells co-cultured with mouse glia. Consistent with this finding, maturation of fibroblast-derived NPCs was enhanced when implanted into embryonic or adult mouse brains [125]. This observation represents one of the most promising future applications of stem cell research: to produce grafts for neurological diseases.

Several studies have transplanted ESC-derived neurons into animal models of neurological diseases. ESC-derived neurons were found to be integrated within brain circuits and to differentiate into mature neurons when implanted into mouse embryos [126]. These neurons exhibited typical neuronal membrane properties associated with postsynaptic currents following electrical stimulation. However, spontaneous synaptic activity was not investigated in this study. In another study, ESC-derived neurons were able to integrate into brain slices [127]. Shortly after implantation (1 to 3 weeks), passive and active membrane properties became neuronal-like, indicating improved maturation compared with *in vitro* studies [9, 10], and spontaneous inhibitory/excitatory postsynaptic currents were present. This trend was confirmed by another study, in which iPSC-derived NPCs were implanted into embryonic mouse brain [76].

With regard to the application of iPSCs for regenerative medicine, the use of iPSCs may eliminate the chances of immune rejection as patient-specific cells may be used for transplantation [128]. There are two main strategies for using iPSCs to treat neurological disorders. The first is to produce new neurons to replace those lost during disease progression. The second strategy is to produce glial cells that could protect neurons from ongoing degeneration by expressing and secreting neuroprotective proteins, such as growth factors [129]. On the other hand, generating neuronal cells from patient-derived tissues allows for the direct investigation of cellular disease-related phenotypes and could lead to the development of suitable treatment options (as reviewed in [130–132]). Additionally, recapitulation of the connectivity and circuitry dysfunction phenotypes in a number of neurological disease-specific iPSCs have been established (summarized in Table 4). Various studies could identify dysfunctional connectivity in neurons generated from patient tissue of various diseases, including AD, amyotrophic lateral sclerosis, Dravet syndrome, Down syndrome, Fragile X syndrome, PD, Phelan–McDermid syndrome, Rett syndrome, SMA and schizophrenia [78, 87–90, 96, 106, 133–145]. For example, the study by Marchetto and colleagues found reduced frequencies of spontaneous synaptic neurotransmission in iPSC-derived neurons of Rett syndrome patients [87]. Similarly, in iPSC-derived neurons from patients with trisomy 21, the frequency of spontaneous neurotransmission was reduced, although it was not specified whether excitatory or inhibitory neurotransmission was affected [90]. In neurons differentiated from Phelan–McDermid syndrome patient-derived iPSCs, excitatory neurotransmission was impaired, as indicated by reduced amplitudes and frequencies of spontaneous excitatory postsynaptic currents [89]. These models will help us to understand the molecular biology underlying diseases and pave the way for re-regulating dysfunctional neuronal circuits. According to safety issues of iPSC therapy, accumulating preclinical evidence supports the effectiveness of iPSC-based cell therapy on the selection of appropriate iPSC clones. Continuous development of safer iPSCs has resulted from insertion-free systems and the use of new transgenes. Nevertheless, before clinical application of iPSC-based cell therapies is achieved, these safety concerns must be assuaged through a thorough examination of the quality of both iPSCs and iPSC-derived cells, in terms of genetic and epigenetic status, differentiation capability both *in vitro* and *in vivo*, and tumorigenicity [146].

**Table 4:** Connectivity and circuitry dysfunction observed in published human iPSC models of neurological diseases

**Disease**	**iPSC-derived cell types**	**Observed phenotypes**	**References**
Alzheimer’s disease	Cortical neurons	• Accumulated extracellular Aβ oligomers inside familial and sporadic neurons, leading to oxidative stress	[78, 133–135]
		• Selectively decreased glutamatergic neurons rather than GABAergic neurons with increasing concentrations of the globulomeric form of Aβ_42_	
		• Redistributed hyperphosphorylated tau to the somatodendritic compartments	
Amytrophic lateral sclerosis	Motor neurons, astrocytes	• Hyperexcitability of amytrophic lateral sclerosis patient-derived motor neurons	[138, 139]
		• Kv7 channel-activator retigabine could revert motor neuron hyperexcitability	
		• Astrocytes from amytrophic lateral sclerosis patient-derived iPSCs show toxicity towards motor neurons in co-culture	
Dravet syndrome	Glutamatergic and GABAergic neurons	• Impaired action potential generation in GABAergic neurons derived from Dravet syndrome patient tissue	[106, 140, 141]
		• Hyperexcitability and spontaneous epileptic action potential firing in glutamatergic neurons	
		• Increased sodium currents	
		• Hyperexcitability was reduced after treatment with phenytoin	
Down syndrome	Cortical neurons	• Defected the ability to form functional synapses in early trisomy of chromosome 21 iPSC neurons	[90, 142]
		• Diminished number of neural progenitor cells associated with a proliferation deficit and increased apoptosis.	
		• Reduced number and length of neurites from soma of neurons	
		• Decreased frequencies of spontaneous neurotransmission, affecting excitatory and inhibitory synapses equally	
Fragile X syndrome	NPCs, neurons of unspecified subtype	• Impaired neuronal differentiation of Fragile X syndrome patient-derived iPSCs	[143, 144]
		• No clear effect on glial differentiation	
		• No activation of mutant FMR1 locus during iPSC generation from Fragile X syndrome patient tissue	
Parkinson’s disease	Dopaminergic neurons	• Reduced numbers of neurites and neurite arborization	[136, 137]
		• Decreased dopamine uptake and disrupted the precision of dopamine transmission by increasing spontaneous dopamine release	
Schizophrenia	Glutamatergic neurons	• Elevated levels of secreted catecholamines including dopamine, norepinephrine, and epinephrine secretion	[88, 91]
		• Increased percentage of tyrosine hydroxylase-positive neurons, the first enzymatic step for catecholamine biosynthesis	
		• Decreased neuronal connectivity and numbers of neurites	
Spinal muscular atrophy	Motor neurons	• Attenuated levels of SMN1 protein in spinal muscular atrophy iPSC neurons, resulting in the selective degeneration of motor neurons	[96, 145]
		• Decreased numbers of motor neuron survival with a reduced size	
		• Reduced axonal growth and neuromuscular junction formation	
Rett syndrome	Glutamatergic neurons	• Diminished number of synapses and dendritic spines	[87]
		• Abnormally decreased activity-dependent calcium oscillations	
		• Reduced frequencies and amplitude of spontaneous synaptic currents, reflecting fewer release sites or a decreased release probability of neurotransmission	
Phelan–McDermid syndrome	Forebrain neurons	• Impaired excitatory neurotransmission indicated by reduced amplitudes and frequencies of spontaneous excitatory postsynaptic currents	[89]
		• Disrupted the ratio of cellular excitation and inhibition in Phelan–McDermid syndrome neurons	

Aβ, amyloid beta; GABA, γ-aminobutyric acid; iPSC, induced pluripotent stem cell; NPC, neural progenitor cell

## Conclusions

Curing neurological diseases is a challenging task in experimental therapeutics. The incidence of neurodegenerative diseases has enormously increased due to a rise in life expectancy. However, there are only symptomatic treatments available for several reasons: a lack of knowledge about disease pathogeneses; limited access to tissue samples for early stages of neurodegeneration; and the inability of animal models to recapitulate all aspects of human diseases. The development of stem cell technology provides a powerful tool in neurobiology that might help solve these problems. The availability of human ESCs, iPSCs and iN cells enables the investigation of diseases in unprecedented depth. Human ESCs can be genetically modified to harbor a desired mutation and develop a phenotype of disease. However, the incomplete penetrance and variability among phenotypes of genetic disease can be an obstacle for accurate disease modeling. Bioethics and limited availability are also of concern. On the other hand, human iPSCs or iN cells might be the best methods available to reprogram patient cells into neurons. They allocate the opportunity to turn back the clock or to uncover the mechanisms underlying cause and progression of sporadic neurodegenerative diseases whose cause has yet to be identified [73]. There is also great expectation for use of stem cell technology in regenerative medicine. For instance, human iPSCs can be prepared from the patient themselves, thereby avoiding graft rejection. Nevertheless, major limitations for transplantation are the delivery of neurons into the appropriate location and the integration of these cells into pre-existing circuits. Other limitations are chromosomal abnormalities leading to clonal variation, teratoma formation, and immaturity of differentiated cells. Thus, iPSC karyotypes need to be tested, and undifferentiated cells should be carefully removed prior to therapeutic application [147]. To this end, maturation protocols that provide iPSC models containing genetic susceptibility in parallel with aging-related factors continue to be developed in order to accurately model late-onset disorders [72].

Electrophysiological techniques are not the only approaches to investigate the phenotype and maturity of stem cell-derived neurons. Genome editing technologies are also being used to demonstrate the relationship between genotype and phenotype. Genome-wide association studies might uncover causative or predisposing genetic loci contributing to multifactorial disease. Another benefit of using stem cell-derived neurons is in large-scale screening of pharmacological agents preventing or curing the disease. The opportunity for personalized medicine is also a very promising application of stem cell technologies in which neurons can be derived from the same individual for whom the therapy is tailored. In summary, recapitulation of human neurological disease following stem cell-derived neuronal differentiation *in vitro* is still a big scientific challenge. Although we can model neuronal circuits in a dish and confirm the expression of neuronal markers and electrophysiological functionality, we cannot yet conclude that these circuits represent the actual connectivity and mature circuitry present throughout the central nervous system. There is thus the need to refine techniques aimed to produce neurons *in vitro* that properly integrate into pre-existing circuits to assume physiological functions [148].

## Abbreviations

AD: Alzheimer’s disease
AP: action potential
C_m_: membrane capacitance
EEG: electroencephalography
ESC: embryonic stem cell
fMRI: functional magnetic resonance imaging
FWHM: full width at half-maximum
GABA: γ-aminobutyric acid
GDP: giant depolarizing potential
iN: induced neuronal
iPSC: induced pluripotent stem cell
LTD: long-term depression
LTP: long-term potentiation
NMDAR: *N*-methyl-d-aspartate receptor
NPC: neural progenitor cell
PD: Parkinson’s disease
R_in_: input resistance
RMP: resting membrane potential
SMA: spinal muscular atrophy
τ: membrane time constant
